# Modeling effective thermal conductivity enhanced by surface waves using the Boltzmann transport equation

**DOI:** 10.1038/s41598-022-19873-3

**Published:** 2022-09-14

**Authors:** Kuk Hyun Yun, Bong Jae Lee, Seong Hyuk Lee

**Affiliations:** 1 School of Mechanical Engineering, Chung-Ang University, Seoul, 06974 Republic of Korea; 2 Department of Mechanical Engineering, Korea Advanced Institute of Science and Technology, Daejeon, 34141 Republic of Korea; 3 Department of Intelligent Energy and Industry, Chung-Ang University, Seoul, 06974 Republic of Korea

**Keywords:** Mechanical engineering, Applied physics

## Abstract

The thermal management of semiconductors at the device level has become a crucial issue owing to the high integration density and miniaturization of microelectronic systems. Because surface phonon polaritons (SPhPs) exhibit long propagation lengths, they are expected to contribute significantly to the heat dissipation in microelectronic systems. This study aims to numerically estimate the heat transfer due to SPhPs in a thin SiO_2_ film. The one-dimensional Boltzmann transport equation (BTE) is solved using the estimated propagation length based on the SPhP dispersion curves. The temperature profiles and heat fluxes are predicted and demonstrate the size effect of the film on the effective in-plane thermal conductivity of the SiO_2_ film. The results indicate that the temperature distribution was constant regardless of the film length and thickness because the propagation length was much longer than the film length. In addition, the heat flux increased with decreasing film thickness owing to the depth-averaged energy transfer. The effective thermal conductivities predicted using the BTE differed by ~ 16.5% from the values obtained from the analytical expression. The numerical results of this study can provide valuable data when studying the thermal behavior of SPhPs.

## Introduction

Surface phonon polaritons (SPhPs) are the collective motion of optical phonons coupled with photons (or electromagnetic waves) in polar dielectrics. Physically, SPhPs can have long propagation lengths of up to several millimeters or even several meters, which is several orders of magnitude greater than their phonon mean free path, along the surface of polar dielectrics^1–3^. Therefore, SPhPs can ballistically transport thermal energy. Many studies have investigated SPhPs as additional thermal energy carriers owing to their long propagation lengths^4–7^. Two decades ago, Chen et al.^8^ first predicted the effective in-plane thermal conductivity of SPhPs (*κ*_*SPhPs*_) in a freestanding SiO_2_ film that is isotropic and homogeneous materials. Their results indicate that *κ*_*SPhPs*_ increases dramatically when the film thickness is less than 100 nm. Recently, Tranchant et al.^9^ and Wu et al.^10^ experimentally demonstrated the variations in *κ*_*SPhPs*_ with the thickness, length, and temperature of a thin film. Owing to their enhanced effective in-plane thermal conductivity, SPhPs are potentially useful for heat dissipation purposes in next-generation microelectronics systems. Currently, the thermal management in microelectronics is mainly conducted at the package level. Nevertheless, device-level thermal management has garnered substantial attention because of their high integration density and miniaturization of microelectronic systems^11,12^. Therefore, predicting the heat transfer capabilities of SPhPs is important; however, their temperature distribution and heat flux have rarely been analyzed.

In this paper, the Boltzmann transport equation (BTE) is used instead of Fourier’s law to predict the temperature profile and heat flux at the nanoscopic scale. It is well known that Fourier’s law cannot predict heat conduction at the submicron scale, where the characteristic length scale is comparable to, or smaller than, the mean free path of the energy carrier^13–16^. Conversely, BTE can describe the non-diffusive (*i*.*e*., ballistic) heat conduction at such a small scale and can consider spatial behavior in anisotropic and inhomogeneous materials or film defects. For example, Majumdar^17^ and Joshi and Majumdar^18^ predicted the temperature distribution within a nanoscale diamond substrate. Specifically, they derived the equation of phonon radiative transfer in terms of the phonon intensity from the BTE using the relaxation time approximation. Chen et al.^19–21^ developed a ballistic-diffusive equation (BDE) based on the BTE to describe the diffusive and ballistic energy transport contributions of the energy carriers and calculated the temperature and heat flux in the imaginary material. These studies show that the BTE can describe nanoscale heat transfer well.

This study aims to quantitatively analyze the heat transfer due to SPhPs in a nanoscale thin film using the BTE and lay the foundation for heat dissipation design considering SPhPs. Specifically, the one-dimensional BTE is established and directly solved to predict the temperature field, heat flux, and effective in-plane thermal conductivity of SPhPs in a freestanding SiO_2_ film. The effects of the length and thickness of the SiO_2_ film on the effective in-plane thermal conductivity of the SPhPs are further examined using the established BTE. The numerical solution to the BTE for the SPhPs in a freestanding SiO_2_ film is compared with the theoretical value obtained by Tranchant et al.^9^

## Mathematical modeling

### Boltzmann transport equation for surface phonon polaritons

**Figure 1:**
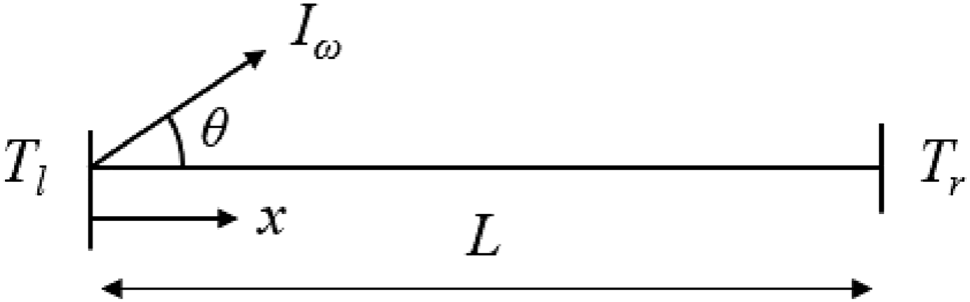
Numerical analysis domain of the one-dimensional BTE.

The one-dimensional BTE is expressed with the relaxation time approximation^22^ as

1 $$\frac{\partial f}{{\partial t}} + v_{g} \cdot \nabla f = \frac{{f_{0} - f}}{\tau },$$

where *f* is the distribution function of the SPhPs, *v*_*g*_ is the group velocity of the SPhPs, *f*_0_ is the equilibrium distribution function (Bose-Einstein distribution function), and *τ* is the relaxation time. Assuming that the film is sufficiently thin such that the heat transfer in the thickness direction can be neglected, the one-dimensional steady-state BTE along the in-plane direction (*x-*direction in Fig. 1) can be rewritten using the intensity notation as

2 $$\cos \theta \frac{{\partial I_{\omega } }}{\partial x} = \frac{{I_{\omega }^{0} - I_{\omega } }}{{\Lambda_{e} }},$$

where Λ_*e*_ is the effective propagation length of the SPhP obtained using Matthiessen’s rule; that is, Λ_*e*_^−1^ = Λ_*SPhP*_^−1^ + *L*^−1^, where Λ_*SPhP*_ is the propagation length of the SPhP and *L* is the film length^1^. In addition, *θ* is the polar angle along the *x*-axis, and ${I}_{\omega }$ and ${I}_{\omega }^{0}$ are the directional SPhP intensity for a particular frequency per unit length (W s m^−1^ rad^−1^) and the equilibrium SPhP intensity, respectively, and are defined as (Supplementary information S1)

3 $$I_{\omega } \left( {x,\omega } \right) = \left| {v_{g} } \right|\hbar \omega f\left( x \right)\,D_{2D} \left( \omega \right)/2\pi$$

4 $$I_{\omega }^{0} \left( {x,\omega } \right) = \frac{1}{2\pi }\int_{0}^{2\pi } {I_{\omega } \left( {x,\omega } \right)d\theta } ,$$

where *ħ* is the Planck constant divided by 2*π*, *ω* is the SPhP frequency, and *D*_*2D*_(*ω*) is the SPhP density of states per unit area. Once the SPhP intensity is determined, the temperature distribution and heat flux (*q*_*SPhPs*_) can be obtained by integrating over the SPhP frequency range as

5 $$\int_{{\omega_{L} }}^{{\omega_{H} }} {I_{\omega }^{0} \left( {x,\omega } \right)d\omega } = \int_{{\omega_{L} }}^{{\omega_{H} }} {\frac{{\left| {v_{g} } \right|}}{2\pi }\frac{{\hbar \omega D_{2D} \left( \omega \right)}}{{\exp \left[ {\hbar \omega /k_{B} T\left( x \right)} \right] - 1}}d\omega }$$

6 $$q_{SPhPs} = \frac{1}{d}\int_{{\omega_{L} }}^{{\omega_{H} }} {\int_{0}^{2\pi } {I_{\omega } \cos \theta \,d\theta } \,d\omega } ,$$

where *ω*_*H*_ and *ω*_*L*_ are the highest and lowest frequencies of the SPhPs, respectively, and *d* is the film thickness. In Eq. (6), the heat flux is inversely proportional to the film thickness. The heat flux of a surface polariton includes the assumption that the amount of in-plane energy transferred by the SPhPs is constant regardless of the position according to film thickness direction and is a depth-averaged value. Because SPhPs can propagate in any direction on the surface of a thin film, the numerical method used to solve the BTE should include the discretization of space and polar angle. In the present study, the finite element method was used with the discrete ordinates method (DOM) to discretize the spatial and angular terms of the BTE. The DOM numerically integrates angles by assigning a weight function *w* to a specific angle^23^. The Gaussian–Legendre quadrature was adopted to integrate in polar angle. The boundary conditions of the BTE were given by the constant equilibrium intensity of the SPhPs at a fixed temperature at the boundary. Because the SPhPs propagate forward (positive *x*-direction) when cos*θ* > 0 and backward (negative *x*-direction) when cos*θ* < 0, the boundary conditions for the two cases can be expressed as^18^

7a $$I_{\omega } \left( 0 \right) = I_{\omega }^{0} \left( {T_{l} } \right)\,\,\,\,\,{\text{when}}\,\,\cos \theta > \,0$$

7b $$I_{\omega } \left( L \right) = I_{\omega }^{0} \left( {T_{r} } \right)\,\,\,\,\,{\text{when}}\,\,\cos \theta < \,0,$$

where *T*_*l*_ and *T*_*r*_ are the temperatures at the boundaries of the analysis domain, as shown in Fig. 1.

### Effective in-plane thermal conductivity

The effective in-plane thermal conductivity due to the SPhPs in the layered thin film is given as follows^8,9^:

8 $$\kappa_{theory} = \frac{1}{4\pi d}\int_{{\omega_{L} }}^{{\omega_{H} }} {\hbar \omega \beta_{R} \Lambda_{e} \frac{{\partial f_{0} }}{\partial T}d\omega } ,$$

where *β*_*R*_ is the real part of the SPhP in-plane wave vector *β*. Equation (8) expresses the effective in-plane thermal conductivity at an interface of infinite length. The effective propagation length is used in consideration of the boundary scattering caused by the propagation length of the SPhPs, which is significantly longer than the film length^1,9^.

The optical phonons are thermally excited in the frequency range of 7.6–258 Trad/s for SiO_2_^9^. In this frequency range, the SPhPs can be classified into three modes: Zenneck, SPhP, and transverse magnetic (TM)-guided modes^24^. However, the TM-guided mode was excluded because it does not satisfy the existence condition of SPhPs on a SiO_2_ film^9^. Therefore, we considered both the SPhP and Zenneck modes in this study.

### Dispersion relation of surface phonon polaritons

The dispersion relation estimates the physical properties of energy carriers such as propagation length and group velocity as a relationship between frequency and wave vector. These properties were required to solve the BTE and obtain theoretical solutions.

**Figure 2:**
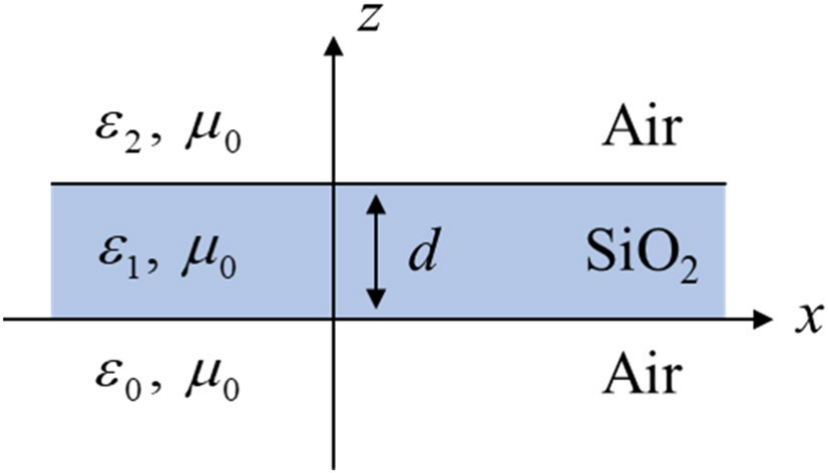
Cross-section of an amorphous SiO_2_ thin film surrounded by air.

The dispersion relation for an amorphous SiO_2_ thin film, assumed to be a nonmagnetic material surrounded by air as shown in Fig. 2, can be derived from Maxwell’s equations for the three layers of the thin film^25^:

9 $$\tanh \left( {\frac{{p_{1} d}}{2}} \right) = - \frac{{p_{0} \varepsilon_{1} }}{{p_{1} \varepsilon_{0} }},$$

where *p*_*j*_ is the transverse wave vector of the SPhPs given by ${p}_{j}^{2}={\beta }^{2}-{\varepsilon }_{j}{k}_{0}^{2}$, *ε*_*j*_ is the relative permittivity of the material (*j* = 0, 1, 2), and *k*_*0*_ is the wave vector defined as the frequency divided by the speed of light in a vacuum.

For lossy dielectric materials, the permittivity can have a complex form and can be expressed as the relationship between the refractive index *n* and the extinction coefficient *k* of the material; that is, *ε* = (*n* + *i∙k*)^2^, where *i* is an imaginary number. The permittivity of amorphous SiO_2_ at room temperature (300 K) was obtained from the experimental data, using its refractive index and extinction coefficient^26^.

## Results and discussion

To consider the propagating nature of the SPhPs along the surface of the SiO_2_ film, the wave vector of the SPhPs was set to a complex number (i.e., *β* = *β*_*R*_ + *i*∙*β*_*I*_) and the frequency was set to a real number. From the dispersion relation, the propagation length of the SPhPs was defined as Λ_*SPhP*_ = 1/(2*∙β*_*I*_)^27^.

**Figure 3:**
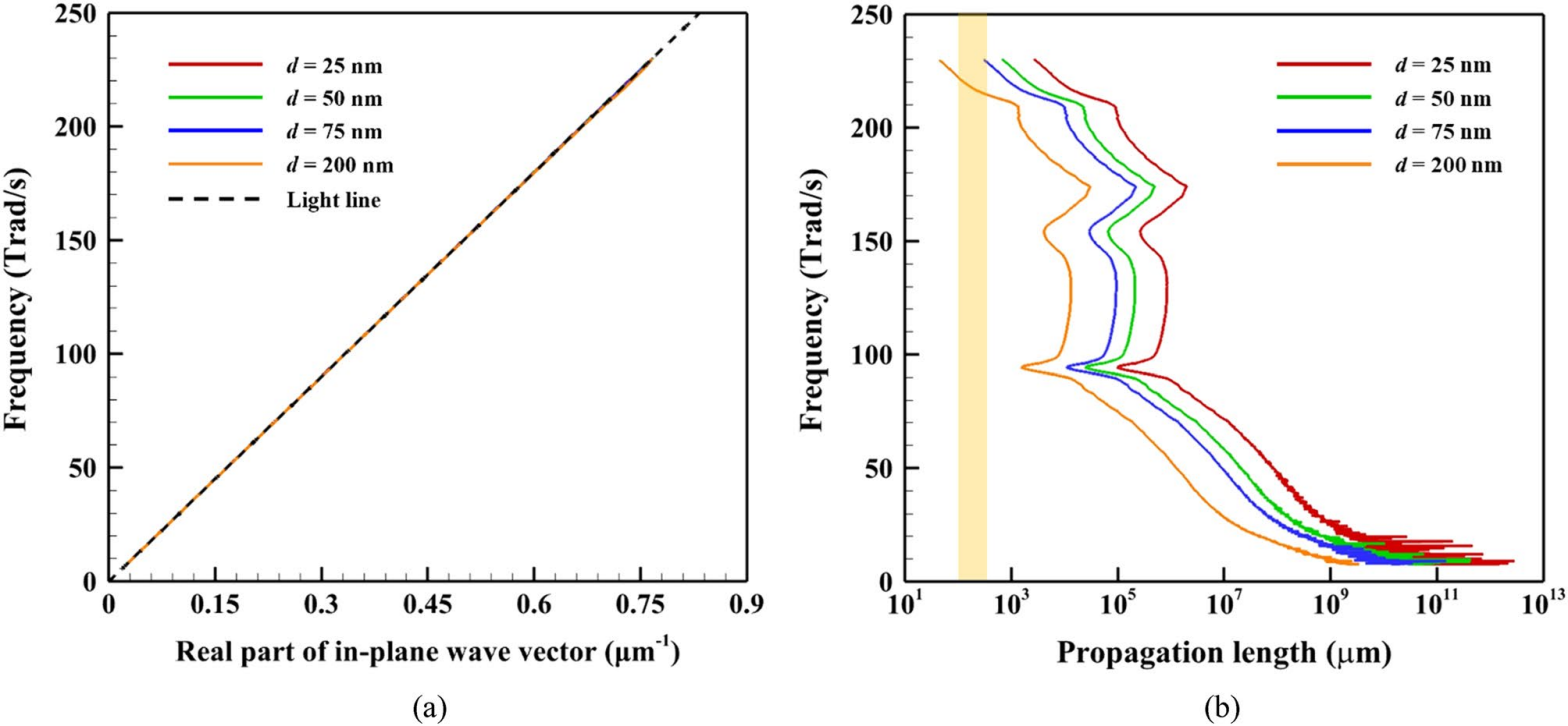
(**a**) Dispersion relation; (**b**) propagation length of the SPhPs on the amorphous SiO_2_ film surface of various thicknesses. The yellow area corresponds to the range of the film length used in this study.

The dispersion relation consists of the frequency and the real part of the wave vector, as shown in Fig. 3a. Because the trend of the dispersion relation describing the SPhPs in SiO_2_ films of different thicknesses aligns with the light line, it can be deduced that the group velocity (*v*_g_ = d*ω*/d*β*_*R*_) of the SPhPs is comparable to the speed of light. Thus, the SPhP density of states per unit area can be expressed as

10 $$D_{2D} \left( \omega \right) = \frac{\omega }{{2\pi v_{g}^{2} }}.$$

Figure 3b shows that the propagation length of the SPhPs on the surface of a freestanding SiO_2_ film increases with decreasing film thickness. In addition, when the film thickness was 25 nm, the SPhPs propagated for up to several meters, particularly at low frequencies. Therefore, the long propagation length of SPhPs is expected to result in high thermal conductivity for thinner films.

In this study, the same SiO_2_ film dimensions used in the report by Tranchant et al.^9^ were applied as a preliminary study to compare the experimental and theoretical results of the effective in-plane thermal conductivity of SPhPs. Thus, the film lengths were 120 μm, 195 μm, 275 μm, and 350 μm, and the film thicknesses were 25 nm, 50 nm, 75 nm, and 200 nm. The BTE was solved for 16 cases with each length and thickness of the film. For all cases, the temperature difference to generate heat transfer was set to ∆*T* = *T*_*l*_ – *T*_*r*_ = 1 K, where *T*_*r*_ was set to 300 K. The BTE was solved using COMSOL Multiphysics 5.6, a commercial package that was employed to compute the BTE in several studies^28,29^.

Grid independence tests were performed for both the angular and spatial discretization for a film length of 120 *μ*m and film thickness of 25 nm by monitoring the heat flux and temperature difference between both ends of the film. The number of Gaussian points and elements ranged between 4–32 and 20–60, respectively. The temperature difference and heat flux gradually converged as the number of Gaussian points increased. The deviations in the temperature difference and heat flux between the 16th and 32nd Gaussian points were 0.077% and 0.053%, respectively. Hence, in this study, the number of Gaussian points was set to 16. Conversely, even if the number of elements increased, the computational time and temperature difference remained; therefore, it was decided that 60 elements would be used.

**Figure 4:**
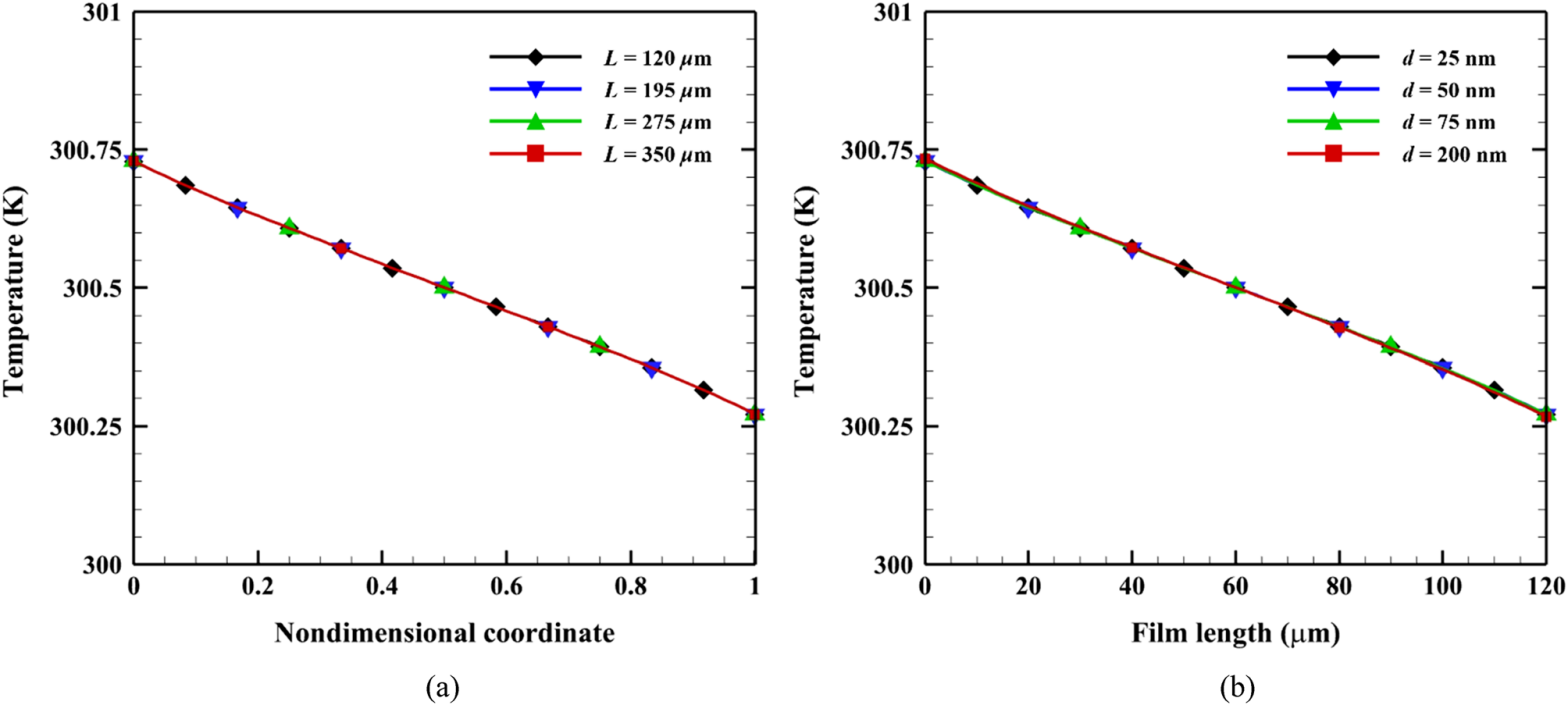
Temperature distribution (**a**) for various film lengths when *d* = 25 nm; (**b**) for various film thicknesses when *L* = 120 µm.

Figure 4 shows the temperature distribution of the freestanding SiO_2_ film for various film lengths and thicknesses. Temperature jumps caused by the ballistic energy transport of the SPhPs were observed at the film boundary for all cases. The temperature distributions were almost identical regardless of the film length and thickness; however, the temperature gradient (∆*T*/*L*) decreased with an increase in the film length, where ∆*T* is the temperature difference. This uniformity is due to the effective propagation length used in the BTE. Since the propagation length of the SPhPs on the SiO_2_ film was significantly greater than the film length, as shown in Fig. 3b, the effective propagation length using Mathiessen’s rule became equal to the film length for most frequencies (Λ_*e*_~*L*). Therefore, the SPhPs exhibited the same behavior as that of boundary scattering for all film-length ranges. For this reason, we expect that there would be temperature differences when the film length is greater than the propagation length for most frequency ranges.

**Figure 5:**
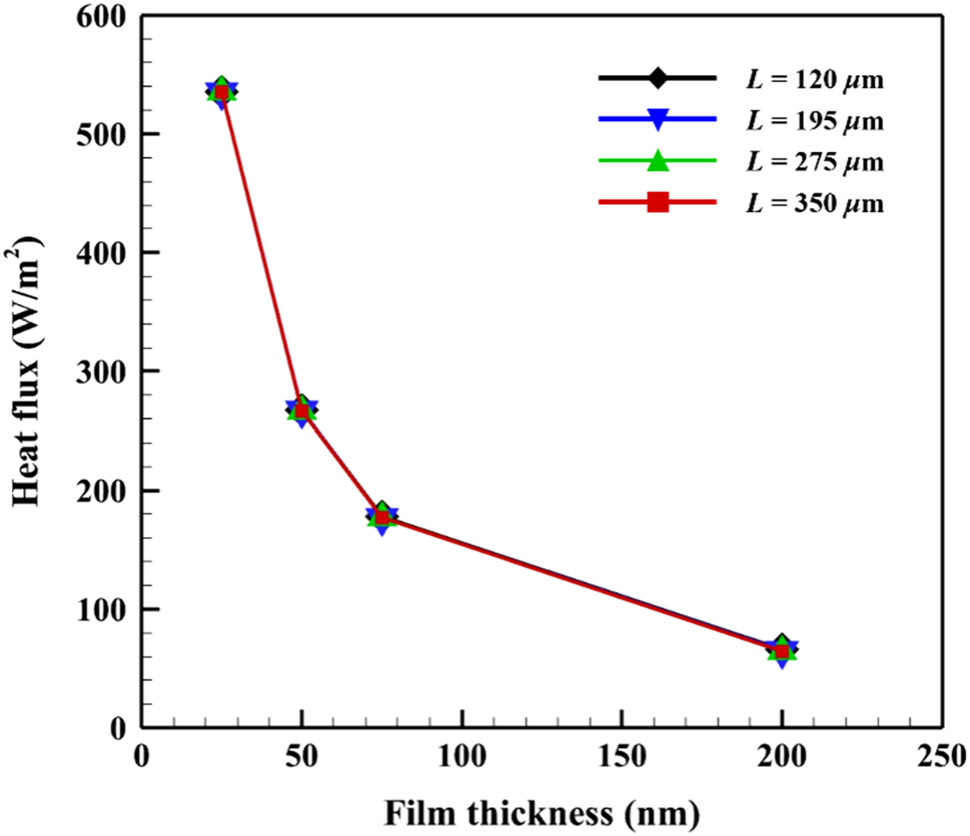
Heat flux of the SPhPs for various film lengths and thicknesses.

Figure 5 shows the effect of the SiO_2_ film thickness on the heat flux of the SPhPs for various film lengths. The heat flux was also nearly the same regardless of the film length, similar to the temperature distribution. However, the heat flux increased with a decrease in the film thickness and reached a maximum value at *d* = 25 nm. The effective propagation length was almost equal to the film length in this study. Therefore, the divergences in the SPhPs intensities calculated by the BTE based on the film length were not large. The heat flux was more affected by the thickness of the film, as described by Eq. (6). The heat flux may differ based on the film length when the effective propagation lengths of the SPhPs are not equal to the film length for most frequency ranges.

**Figure 6:**
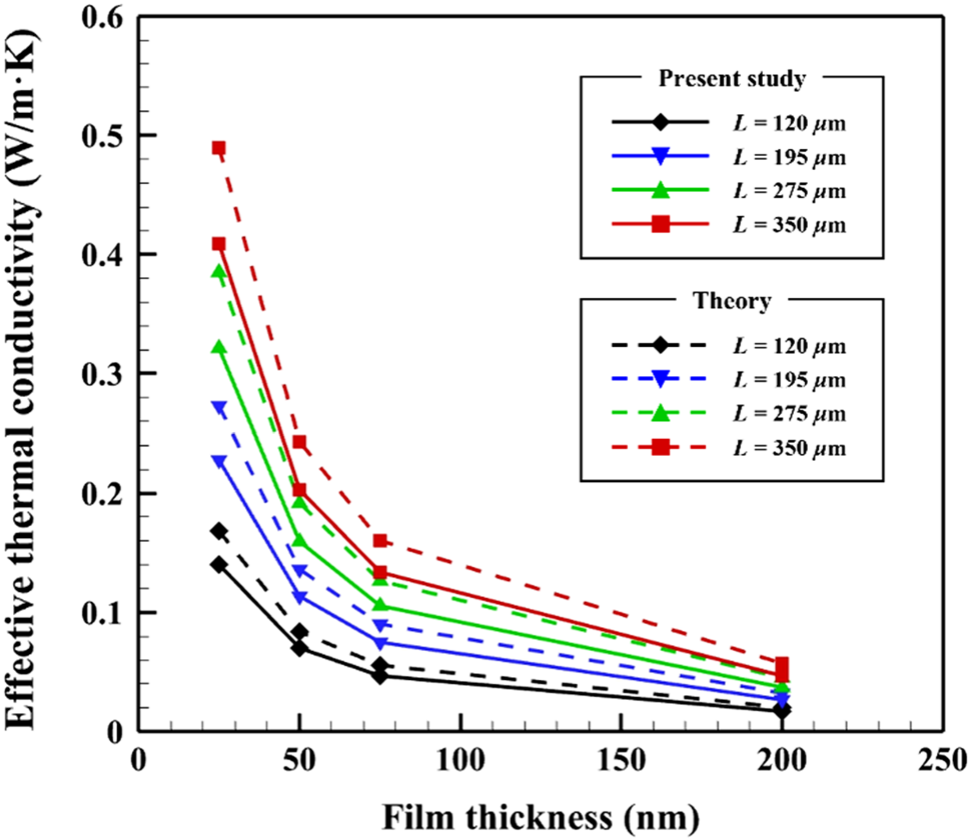
Effective in-plane thermal conductivity due to the SPhPs on the SiO_2_ film. Solid and dashed lines represent the values obtained from the BTE and theory^9^, respectively.

The effective in-plane thermal conductivity is defined as *κ*_*BTE*_ = *q*_*SPhPs*_∙*L*/∆*T*, based on Fourier’s law. We predicted the effective in-plane thermal conductivity using the temperature distribution and heat flux calculated from the numerical solution to the BTE, as shown in Fig. 6 (solid line). The effective in-plane thermal conductivity increased as the film thickness decreased and the length increased. This is because the gradient of the temperature distribution decreases with increasing film length and the heat flux increases with decreasing film thickness. The predicted maximum effective in-plane thermal conductivity was 0.409 W/m K, ~ 29.2% of the thermal conductivity (1.4 W/m K) of phonons in bulk amorphous SiO_2_ when *L* = 350 *µ*m and *d* = 25 nm. Zenneck modes contribute more to the enhancement of the effective thermal conductivity among surface waves as reported in a previous study^9^. Since the same frequency range was used in the numerical method, the effective thermal conductivity was also enhanced by the Zenneck modes. The enhancement in the thermal conductivity clearly indicates that the surface waves at both the SiO_2_-air interfaces can be useful for heat dissipation purposes in microelectronics.

Figure 6 also compares the calculated effective in-plane thermal conductivity with the theoretical values (see Eq. (8)). The effective in-plane thermal conductivity calculated by the BTE exhibits the same tendency as that of the values obtained from theory, but its value is always less by ~16.5% for all cases. It is worth noting that we directly solved the original form of the BTE in Eq. (2). In contrast, Tranchant et al.^9^ considered that only a diffusive part of the SPhPs would contribute to the thermal conductivity; that is, they calculated the effective in-plane thermal conductivity based on the diffusion approximation, ∂*f*/∂*x* ≈ ∂*f*_0_/∂*x*.

## Conclusions

In this study, the BTE for SPhPs was numerically solved to investigate the ballistic energy transport due to the SPhPs on the surface of an amorphous SiO_2_ thin film. In particular, the dispersion relation of SPhPs on an amorphous SiO_2_ film was used to obtain the propagation length and group velocity of the SPhPs for different film thicknesses and lengths. The film temperature distribution and heat flux were estimated based on the SPhP intensity. The conclusions of this study are as follows:

1. From the dispersion relation, it was found that the SPhPs on the amorphous SiO_2_ film surrounded by air had long propagation lengths of several meters. It was confirmed that the SPhPs with long propagation lengths were the energy carriers that were conducive to ballistic energy transport;
2. The temperature distribution was almost identical regardless of the film length and thickness because the effective propagation length was equal to the film length for most frequency ranges and exhibited temperature jumps owing to the ballistic energy transport due to the SPhPs. However, the heat flux increased with the decrease in the film thickness owing to the depth-averaged energy transfer;
3. The effective in-plane thermal conductivity of the SPhPs increased with increasing film length and decreasing film thickness. In addition, the effective thermal conductivity calculated by the BTE was ~ 16.5% lower than the theoretical results. The current numerical approach can provide useful information regarding the thermal characteristics of SPhPs.

For further studies, the BTE should be solved for conditions under which the film length is greater than the propagation length of the SPhPs. In addition, the numerical solution to the BTE can be readily extended to 2-D simulations, and SPhP-enhanced thermal conductivity in a more complicated geometry can thus be considered by the proposed method. The prediction of the temperature distribution and effective in-plane thermal conductivity due to the SPhPs is expected to be useful in designing various products, such as semiconductors, in microelectronics.

## Supplementary Information

Supplementary Information.

## Abbreviations

*c*: Speed of light in a vacuum, m s^−^^1^
*d*: Film thickness, m
*D*_*2D*_(*ω*): SPhP density of states, m^−2^ s
*f*: Distribution function of SPhP
*f*_0_: Equilibrium distribution function of SPhP
${I}_{\omega }$: Directional SPhP intensity, W m^−1^ rad^−1^ s
${I}_{\omega }^{0}$: Equilibrium SPhP intensity, W m^−1^ rad^−1^ s
*k*: Extinction coefficient
*k*_*0*_: Wave vector of light in a vacuum, m^−^^1^
*L*: Film length, m
*n*: Refractive index
*p*: Transverse wave vector of SPhP, m^−1^
*q*_*SPhPs*_: Heat flux of SPhPs, W m^−2^
*t*: Time, s
*T*: Temperature, K
*T*_*l*_: Left boundary temperature, K
*T*_*r*_: Right boundary temperature, K
*v*_*g*_: Group velocity of SPhPs, m s^−1^
*w*: Weight function of Gaussian–Legendre quadrature

## Greek symbols

*β*: SPhP in-plane wave vector, m^−1^
*β*_*R*_: Real part of the SPhP in-plane wave vector, m^−1^
*β*_*I*_: Imaginary part of the SPhP in-plane wave vector, m^−1^
*ε*: Relative permittivity of a dielectric material
*ħ*: Planck’s constant divided by 2π, J s
*θ*: Polar angle, radians
*κ*_*thoery*_: Effective in-plane thermal conductivity obtained from theory, W m^−1^ K^−1^
*κ*_*BTE*_: Effective in-plane thermal conductivity obtained from BTE, W m^−1^ K^−1^
Λ_*e*_: Effective propagation length of SPhP, m
Λ_*SPhP*_: Propagation length of SPhP, m
*τ*: Relaxation time, s
*ω*: SPhP frequency, rad s^−1^
*ω*_*H*_: Highest SPhP frequency, rad s^−1^
*ω*_*L*_: Lowest SPhP frequency, rad s^−1^
