# Supplementary material for: Modeling effective thermal conductivity enhanced by surface waves using the Boltzmann transport equation
Source: Sci Rep. 2022 Sep 14;12:15477. doi: 10.1038/s41598-022-19873-3 (PMC9474515; doi:10.1038/s41598-022-19873-3)
Supplement: Supplementary file 1 — Supplementary Information. [file 41598_2022_19873_MOESM1_ESM.docx]

Modeling effective thermal conductivity enhanced by surface waves using the Boltzmann transport equation

# Kuk Hyun Yun1 , Bong Jae Lee2,*, Seong Hyuk Lee1,3,*

1Chung-Ang University, School of Mechanical Engineering, Seoul, 06974, Republic of Korea

2Korea Advanced Institute of Science and Technology, Department of Mechanical Engineering, Daejeon, 34141, Republic of Korea

3Chung-Ang University, Department of Intelligent Energy and Industry, Seoul, 06974, Republic of Korea

*[bongjae.lee@kaist.ac.kr](mailto:bongjae.lee@kaist.ac.kr); [shlee89@cau.ac.kr](mailto:shlee89@cau.ac.kr)

# Boltzmann transport equation for surface polaritons from a numerical approach

The heat flux of surface polaritons (SPs) is given by^1,2^

, (1)

where *a*, *b*, and *d* are the width, length, and thickness of the film, respectively; ℏ is the Planck constant divided by 2*π*; *ω* is the frequency of SPs; *f* is the distribution function; and *v_g_* is the group velocity of the SPs, with *k_x_* and *k_y_* being their wave vectors. Further, Eq. (1) can be expressed in the integral form as follows:

, (2)

where *θ* is the polar angle along the *x*-axis. Furthermore, we can rewrite Eq. (2) in polar coordinates as

, (3)

where *k*_//_ is the in-plane wave vector. Using the two-dimensional density of states (per unit area) for the SPs, that is, *D_2D_*(*ω*) = *k*_//_/2*π* ∙ (d*k*_//_/d*ω*), Eq. (3) can be modified as follows:

. (4)

Here, we can define the intensity and equilibrium intensity of the SPs (W·s·m^-1^ rad^-1^) for a particular frequency as

 (5)

$I_{\omega}^{0}=\frac{1}{2\pi}\int_{0}^{2\pi} I_{\omega}d\theta$, (6)

where 2*π* indicates a particular direction in Eq. (5) and the average polar angle of intensity in Eq. (6). Additionally, Eq. (4) can be written using the intensity notation as

$q''_{x}=\frac{1}{d}\int_{\omega_{L}}^{\omega_{H}} \int_{0}^{2\pi} I_{\omega}\cos\theta d\theta d\omega$, (7)

where *ω_H_* and *ω_L_* denote the highest and lowest frequencies, respectively, of the SPs. Equation (7) represents the heat flux of the SPs, which is inversely proportional to the film thickness. This implies that the SPs, which transfer heat in the in-plane direction, transmit uniform energy in the film thickness direction. Therefore, the heat flux was significantly affected by the film thickness.

# Theoretical effective thermal conductivity with diffusion approximation

In the heat flux equation obtained from the numerical approach, the distribution function of the SPs was maintained. However, theoretically, the distribution function can be expressed using the Boltzmann transport equation based on the diffusion approximation (∂*f*/∂*x* ≈ ∂*f*_0_/∂*x*, ∂*f*/∂*y* ≈ ∂*f*_0_/∂*y*)^2^, that is,

$f=f_{0}-\Lambda\cos\theta\frac{\partial f_{0}}{\partial x}-\Lambda\sin\theta\frac{\partial f_{0}}{\partial y}$, (8)

where *f_0_* is the equilibrium distribution function of the SPs and Λ is their propagation length. Using Eq. (8), the definition of group velocity (*v_g_* = d*ω*/d*k*_//_), and polar coordinate notation, we can rewrite Eq. (3) as

. (9)

In Eq. (9), the integral of the polar angle can be easily calculated. Thereafter, Eq. (9) can be rewritten as follows:

, (10)

Subsequently, using Fourier’s law in Eq. (10), we can obtain the effective in-plane thermal conductivity of the SPs, *κ_eff_*, as follows:

. (11)

In Eq. (11), the gradient of the temperature of the equilibrium distribution function has been derived from the diffusion approximation. The diffusion approximation assumes that only the diffusive part of the SPs contributes to the thermal conductivity. Furthermore, this approximation replaces the non-computable distribution function with an equilibrium distribution function that can be calculated (Bose–Einstein distribution function). Therefore, the difference in heat flux between the numerical and theoretical approaches caused a constant difference in the effective thermal conductivity.

# References

# Chen, D.-Z. A., Narayanaswamy, A. & Chen, G. Surface phonon-polariton mediated thermal conductivity enhancement of amorphous thin films. *Phys*. *Rev*. *B*. 72, 155435 (2005).

# Tranchant, L. et al. Two-dimensional phonon polariton heat transport. *Nano* *Lett*. 19, 6924-6930 (2019)
